# Applying an innovative biodegradable self-assembly nanomicelles to deliver α-mangostin for improving anti-melanoma activity

**DOI:** 10.1038/s41419-019-1323-9

**Published:** 2019-02-15

**Authors:** Shuping Yang, Xiang Gao, Yihong He, Yuzhu Hu, Bocheng Xu, Zhiqiang Cheng, Mingli Xiang, Yongmei Xie

**Affiliations:** 1 Department of Neurosurgery and Institute of Neurosurgery, State Key Lab of Biotherapy and Cancer Center, West China Hospital, Sichuan University and Collaborative Innovation Center for Biotherapy, Chengdu, 610041 PR China; 2 Department of Pharmacology and Molecular Sciences, Johns Hopkins University School of Medicine, Baltimore, MD 21205 USA

## Abstract

α-Mangostin (αM), a traditional natural product with promising application of treating a series of diseases, was limited use in clinical due to its hydrophobicity. Herein, MPEG-PCL nanomicelles were used to embed the αM for resolving hydrophobicity and improving the anti-melanoma effect of the αM. The anti-melanoma activity and potential mechanisms of biodegradable αM/MPEG-PCL nanomicelles were investigated. The αM/MPEG-PCL nanomicelles possessed a stronger effect on anti-melanoma compared to the free αM both in vitro and in vivo with a low cytotoxicity in non-tumor cell lines. In the research of mechanisms, the αM/MPEG-PCL nanomicelles inhibited the proliferation of melanoma cell, induced apoptosis via both apoptosis pathways of intrinsic and exogenous in vitro, as well as suppressed tumor growth and restrained angiogenesis in vivo, which implied that the αM/MPEG-PCL nanomicelles have potential application as a novel chemotherapeutic agent in melanoma therapy.

## Introduction

Nanotechnology, an international gold rush, is a revolutionizing and rapidly evolving technology inspiring many areas in this word^1,2^. The progress and exploitation of nanotechnology in medicine have conquered tremendous breakthrough in human health career^3,4^. Definitely, the effective nanotechnology is of great significance in the improvement of nanomedicine which has received extensive attention in application of gene therapy, personalized treatments, and precision medicine for oncotherapy^5–7^, notably the development of new drug delivery systems in cancer treatment. At present, several drug delivery systems have been reported, including solid dispersion, multistage drug delivery systems (Ms-DDS), phospholipid complex and so forth^8–10^. However, many defects exist in terms of stability, effectiveness, and preparation. Nanotechnology is a new technique for the application of nanocarriers to encapsulate hydrophobic drugs to improve the water solubility. Nanometer medicine carriers have been diversely developed, such as liposomes, lipids, nanomicelles, silica, and iron oxide. Among them, the applications of biodegradable nanomicelles have received comprehensive attention^11–13^. It is a novel technique for the application of nanocarriers to encapsulate hydrophobic drugs to improve its water solubility and strengthen efficacy^12,13^.

Notoriously, the most devastating form of skin cancer is melanoma with a highly metastatic potential^14,15^. The rate of annual increment is obvious in the last 30 years in the USA, meanwhile, melanoma possesses rapidly increasing incidence rate in whole-world^16^. In 2017, there were 87,110 new cases of melanoma and 9730 deaths associated with the melanoma in the USA^17^. The melanomas possess the highest mutational load in all human cancers, because there are hundreds to thousands of mutations per tumor^18^. However, it is difficult to find a satisfactory therapeutic regimen to treating the melanoma. The major allure of nanomedicine is the possibility of faster and more advanced routes to the settlement of the most pressing and the hardest medical problems. Therefore, one of the more promising ways to treat melanoma is the application of nanomedicine.

**Fig. 1:**
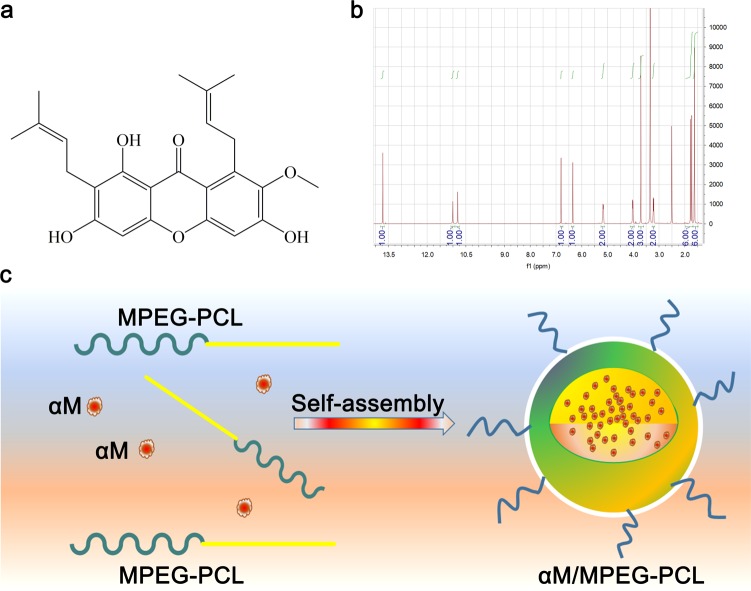
The chemical information of the αM and preparation of the αM/MPEG-PCL micelles. **a** The chemical structure of the αM. **b** ^1^HNMR of the αM. **c** Preparation scheme of the αM/MPEG-PCL nanomicelles. The free αM and the MPEG-PCL self-assembled into the αM/MPEG-PCL nanomicelles

As an exotic fruit, mangosteen was found in South Africa and its peel had used for centuries as a traditional medicine to cure inflammation, ulcer, skin infection, wound healing, and other diseases^19^. α-Mangostin (αM) (Fig. 1a), the major xanthone derivative, is the most important secondary metabolite extracted from the pericarp of mangosteen^19–21^. Meanwhile, the αM was reported with several significant biological activities to benefit health promotion, such as anti-tumor, anti-inflammatory, anti-virus, anti-bacterial, anti-fungal, antioxidant, heart protection, and so on^22–25^. However, the αM is a strong oleophilic compound with poor aqueous solubility^25^, which limits its clinical applications.

In this study, we selected biodegradable nanomicelles of monomethoxy poly (ethylene glycol)-polycaprolactones (MPEG-PCLs) to wrap the αM to improve its water solubility. More significantly, we investigated the efficiency of the αM/MPEG-PCL nanomicelles on the therapy of the melanoma in vitro and in vivo and explored the potential anti-tumor mechanisms.

## Results

### Preparation of the αM/MPEG-PCL nanomicelles

The αM nanomicelles were prepared by a self-assembly method to improve the water solubility. Briefly, the αM and the MPEG-PCL were co-dissolved in anhydrous methanol. After that, the solution was evaporated in rotary evaporator to promote the αM distributed into the MPEG-PCL copolymer. The mixture was dissolved in normal saline solution, which allowed the αM self-assembled into the MPEG-PCL nanomicelles. As shown schematically in Fig. 1c, the αM/MPEG-PCL possessed a core-shell structure with PCL (the hydrophobic segment) as a core to collect αM, and MPEG (the hydrophilic segment) as a shell in aqueous solution.

### Molecular modeling study of interaction between the αM and MPEG-PCL copolymer

**Fig. 2:**
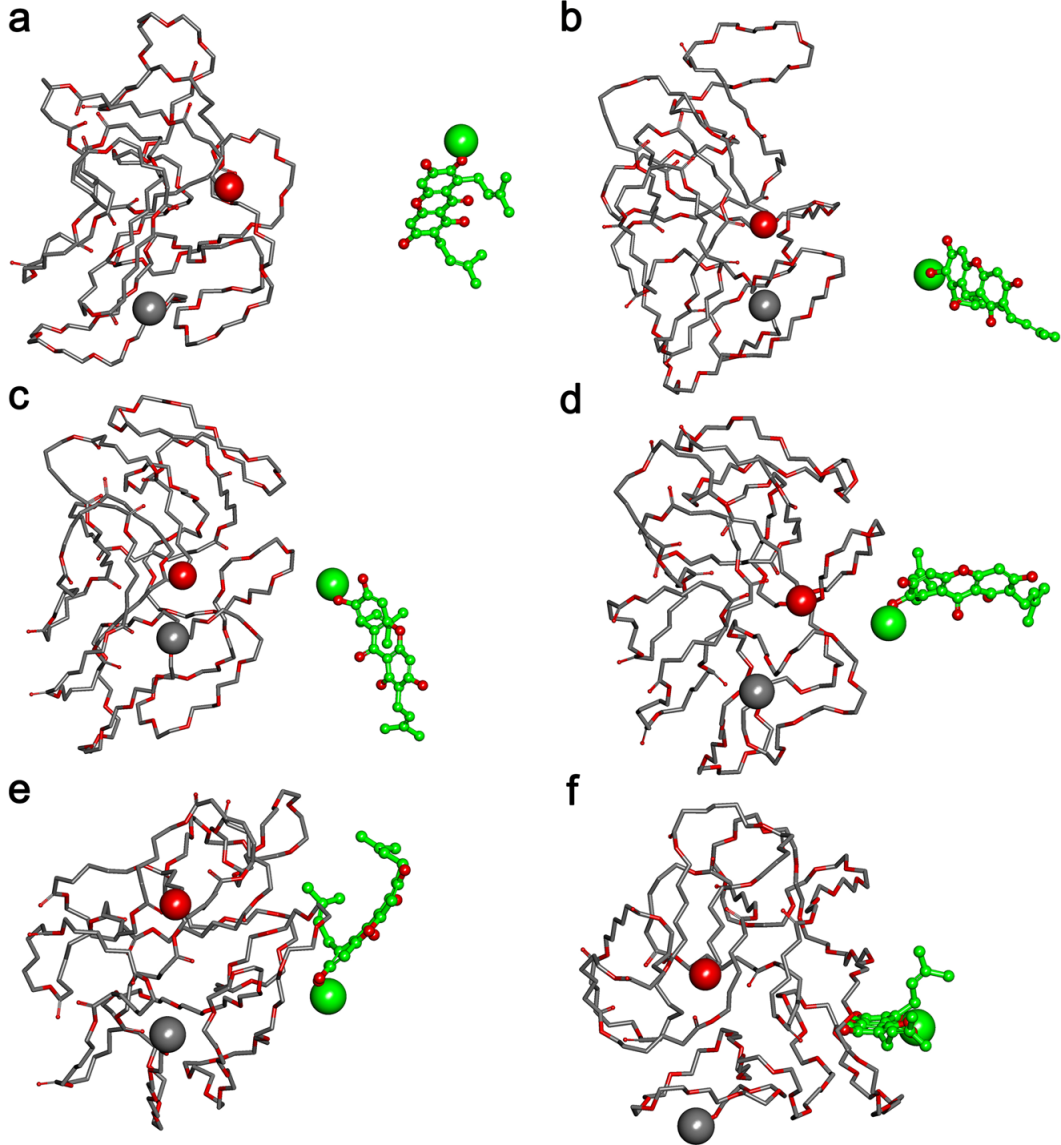
Interaction modes between polymer and compound revealed by Langevin dynamics simulation in an aqueous environment. **a** The initial conformation of the polymer MPEG-PCL complexed with the αM; Conformations **b**, **c**, **d**, **e**, and **f** are corresponding to snapshots of the complex collected at 9.985 ps, 15.995 ps, 20.020 ps, 29.945 ps, and 100 ps, respectively. The polymer MPEG-PCL is represented with thick stick, whereas the αM is depicted with ball and stick style and its carbon atoms are colored with green. Two terminal heavy atoms in the polymer MPEG-PCL is highlighted using a “ball” style, and the head heavy atom in the αM are highlighted using CPK style

As demonstrated in Fig. 2, both the αM and the copolymer MPEG-PCL were trying to adjust their conformations and shorting the distance between them so as to gain a favorable interaction mode.

**Fig. 3:**
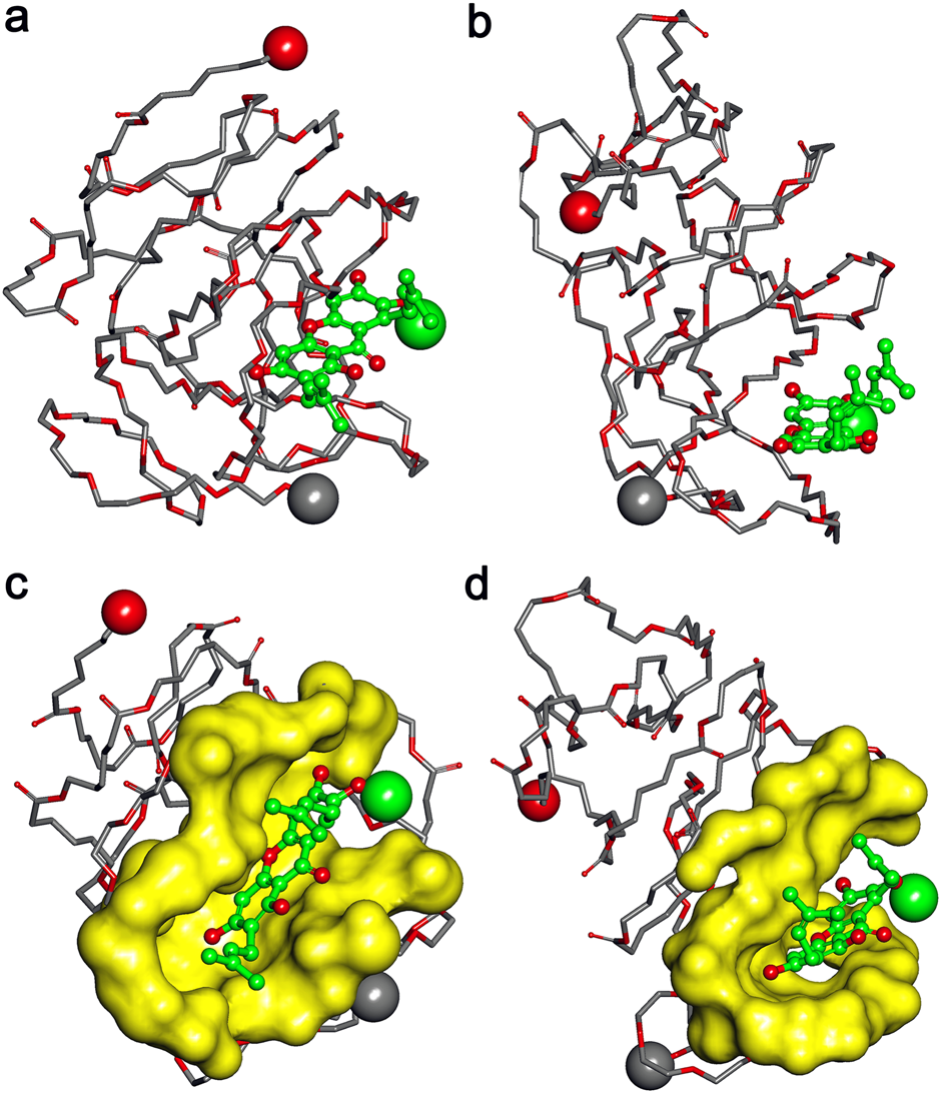
Interaction modes between polymer and compound revealed by Langevin dynamics simulation in water and tumor environment. The demonstration style is the same as in Fig. 2. **a** The conformation of the polymer MPEG-PCL complexed with the αM after being simulated in water environment for 200 ps; **b** The conformation of the polymer MPEG-PCL complexed with the αM after being simulated in water environment for 100 ps and then in tumor environment for 100 ps; **c** and **d** are corresponding to **a** and **b**, but the binding site is highlighted with solid surface and colored light yellow

An interesting result was observed by comparing the simulation of the complex in water and the simulation in tumor. It was shown in Fig. 3 that in these two kinds of different environment, the interaction modes between the αM and the MPEG-PCL were different. In the aqueous environment, copolymer in the αM/MPEG-PCL complex tended to be a globe shape and the binding site was more suitable for interaction with the αM. While in tumor environment, the copolymer seemed not to keep a globe shape and the mouth of the binding pocket was more open that must be easier for the αM to escape from a pocket. In another word, the interaction between the αM and the MPEG-PCL in tumor was weaker than that in water.

### Characterization of the αM/MPEG-PCL nanomicelles

**Fig. 4:**
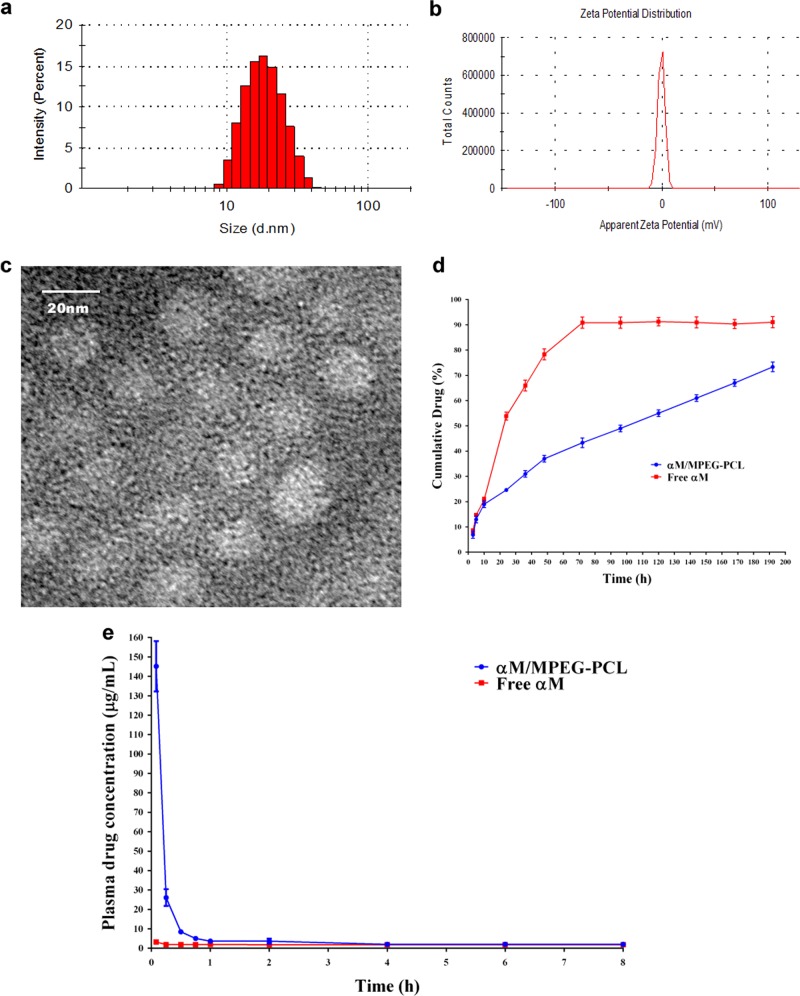
Characterization of the αM/MPEG-PCL nanomicelles. **a** Particle size distribution extent of the αM/MPEG-PCL nanomicelles. **b** Zeta potential spectrum of the αM/MPEG-PCL nanomicelles. **c** The image of transmission electron micrograph (TEM) of the αM/MPEG-PCL nanomicelles. **d** In vitro release assay of the free αM and the αM/MPEG-PCL nanomicelles in PBS containing Tween 80 (0.5%, w/w) at 37 °C. **e** In vivo pharmacokinetics study of the free αM and the αM/MPEG-PCL nanomicelles

The average particle size, polydispersity index, zeta potential, and appearance of αM/MPEG-PCL nanomicelles were further determined and presented. In Fig. 4a, the size distribution spectrum of the freshly prepared αM/MPEG-PCL nanomicelles showed a very narrow particle size distribution (mean particle size = 30 nm) as a monodisperse system. According to Fig. 4b, the αM/MPEG-PCL nanomicelles had a zeta potential of −2.1 mv. Furthermore, transmission electron microscopy (TEM) was applied to study the morphology of the αM/MPEG-PCL, which revealed the αM/MPEG-PCL nanomicelles were spherical with an average diameter of 23 nm in drying phase (Fig. 4c). High-performance liquid chromatography (HPLC) assay illustrated that αM/MPEG-PCL nanomicelles had the drug loading (DL) of 99.1% and encapsulation efficiency (EE) of 10%.

### In vitro release analysis

The in vitro release results indicated the αM was released more slowly from the αM/MPEG-PCL nanomicelles compared to the free αM via dialysis under sink condition with a 0.5% Tween 80 (w/w) solution in phosphate-buffered saline (PBS, pH = 7.4). As shown in Fig. 4d, the αM/MPEG-PCL nanomicelles indicated a much slower cumulative release rate compared to the free αM. During the first 24 h, only 25.3 ± 2.48% of the αM was released into the media in the nanomicelles group, whereas 53.8 ± 0.85% of the αM was released in the free-drug group. At the 72-h point, 91.5% ± 2.55 and 43% ± 0.88% of the αM were released from the free-drug group and the αM/MPEG-PCL nanomicelles, respectively. Therefore, the αM/MPEG-PCL nanomicelles demonstrated a sustained drug-release profile compared with the free drug.

### In vivo pharmacokinetics analysis

Bioavailability improvement by increasing hydrophilicity is the major objective of the encapsulation of the αM into the MPEG-PCL nanomicelles. To prove this hypothesis, we analyzed the mean plasma concentrations of the αM after the tail intravenous injection of the free drug and the nanomicelles drug. As presented in Fig. 4e, the free αM group was fleetly cleared with a plasma level of the αM of 3.26 ± 0.44 μg/mL compared with 145.34 ± 35.56 μg/mL in the group of the αM/MPEG-PCL nanomicelles after injection over 5 min. Apparently, the tremendous difference existed after the 2 h incubation, which proved that the MPEG-PCL nanomicelles significantly reduced the αM elimination.

### Effects on the cell proliferation of melanoma cells and non-tumor cell lines

**Fig. 5:**
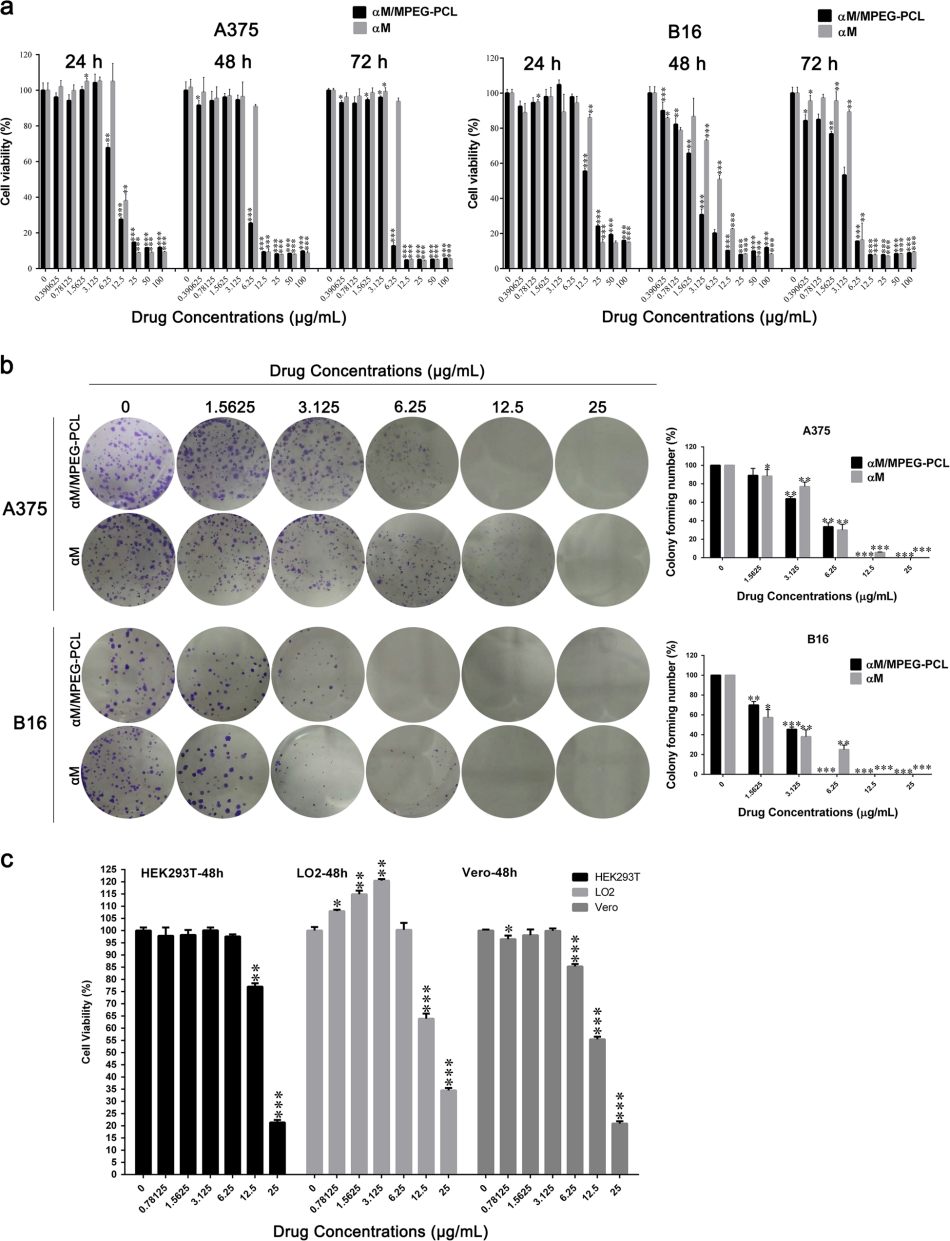
Effects of the free αM and the αM/MPEG-PCL nanomicelles on the cell proliferation. Cell viability was measured with MTT assay. **a** Cell viability of melanoma cells treated with various concentrations of the free αM and the αM/MPEG-PCL nanomicelles for 24 h, 48 h, and 72 h. Values represent mean ± SD (*n* = 3, in triplicate, *P < 0.05; **P < 0.01; ***P < 0.001 compared to control). **b** The effects of the free αM and the αM/MPEG-PCL nanomicelles on colony formation in melanoma cells and the statistic assay of colony formation experiments. Data are expressed as mean ± SD (from three independent experiments), *P < 0.05; **P < 0.01; ***P < 0.001 compared to vehicle (0 μM group). **c** Non-tumor cell lines (LO2, Vero, and HEK293T) were incubated with a series of concentrations of the αM/MPEG-PCL nanomicelles for 48 h. Values represent mean ± SD (*n* = 3, in triplicate, *P < 0.05; **P < 0.01; ***P < 0.001 compared to control)

The anti-cancer activities of the αM and the αM/MPEG-PCL nanomicelles were studied by 3-(4,5-dimethylthiazol-2-yl)−2,5-d/iphenyltetrazolium bromide (MTT) assay. Briefly, A375 and B16 melanoma cells were treated with gradient concentrations of the αM and αM/MPEG-PCL nanomicelles from 0 to 100 μg/mL for 24 h, 48 h and 72 h, respectively. As shown in Fig. 5a, in the lower concentrations, both the αM and αM/MPEG-PCL nanomicelles exhibited no significant inhibition on cell viability of A375 and B16 cells. Subsequently, the suppression effects of the αM and the αM/MPEG-PCL nanomicelles had no obvious dose dependent in the higher concentrations. However, the αM/MPEG-PCL nanomicelles possessed stronger inhibitory effect compared to the αM. In the effectual concentrations, the cell viability of A375 and B16 cells treated with the αM/MPEG-PCL nanomicelles were weaker than the one treated with the αM. These results indicated that the embedding of MPEG-PCL improved the cytotoxicity of the αM to the melanoma cells. To further validate and compare the growth inhibitory effect of the αM and the αM/MPEG-PCL nanomicelles. A375 and B16 cells, clonogenic assay was performed. As shown in Fig. 5b, with the increasing concentrations of the αM and the αM/MPEG-PCL nanomicelles, the size of the colony formation of the melanoma cells obviously reduced. Moreover, no colony formation was observed in A375 cells plate treated with 12.5 μg/mL, as well as B16 cells plate treated with 6.25 μg/mL in the αM/MPEG-PCL nanomicelles group, nevertheless, several colony formations under the same condition were formed in the αM parallel experiments. These results further demonstrated the αM/MPEG-PCL nanomicelles had a more intense restrain effect on the level of colony formation in melanoma cells compared to the αM.

Meanwhile, non-tumor cell lines, including LO2, Vero, and HEK293T cells, were treated by the same condition. The MTT results demonstrated the αM/MPEG-PCL nanomicelles (<12.5 μg/mL) exhibited low a cytotoxicity for non-tumor cell lines in Fig. 5c. Indeed, the cell viabilities were more than 50% and 20%, respectively, when it was treated by 12.5 μg/mL and 25 μg/mL the αM/MPEG-PCL nanomicelles.

### The mechanisms of the anti-melanoma effect of the αM/MPEG-PCL nanomicelles in vitro

**Fig. 6:**
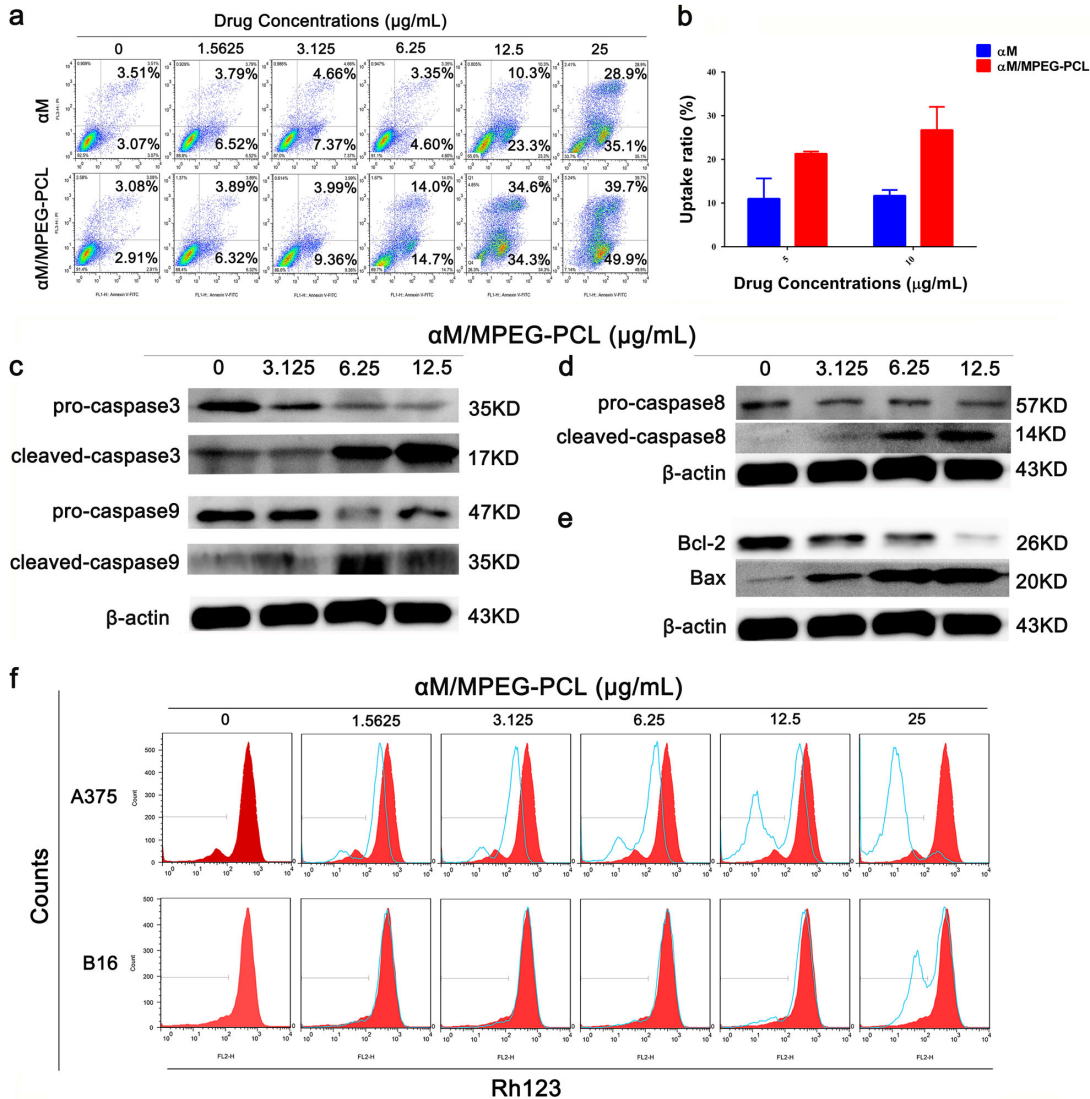
Apoptosis-induced effect and cellular uptake in A375 cells by the free αM and the αM/MPEG-PCL nanomicelles, as well as the mechanisms of the αM/MPEG-PCL nanomicelles anti-melanoma in vitro. **a** PI and Annexin V dual-labeling technique analyzed the apoptotic effect in A375 cells, the αM/MPEG-PCL nanomicelles showed higher cell apoptosis rates than the free αM. **b** A375 cells uptake drug assay. The αM/MPEG-PCL nanomicelles group’s cells uptake almost twice as much as the free αM group. **c** The variation in the mitochondrial membrane potential (Ψm) in melanoma cells by the αM/MPEG-PCL. **d** Upregulation of Bax (pro-apoptosis protein) and downregulation of Bcl-2 (anti-apoptosis protein) in A375 cells treated the αM/MPEG-PCL. **e** The variation of caspase-3 and caspase-9 protein level were determined via western Blot in A375 cells. The results showed that the αM/MPEG-PCL could induce A375 cells apoptosis by activating caspase-3 and caspase-9. **f** The caspase-8 protein level associated with the exogenous apoptosis pathways were altered by the αM/MPEG-PCL micelles in A375 cells. β-actin was used as an internal standard in **d**, **e**, and **f**

To study the apoptosis efficiency induced by the αM and the αM/MPEG-PCL nanomicelles, A375 cells were studied by flow cytometry (FCM) using Annexin V-FITC/PI staining. According to Fig. 6a, the A375 cells apoptosis rates were calculated to 6.58% (free αM group) and 5.99% (αM/MPEG-PCL nanomicelles group) in the negative control. However, in the αM and the αM/MPEG-PCL nanomicelles groups, the cell apoptosis rates were enhanced with the increasing of concentrations of the αM and the αM/MPEG-PCL nanomicelles. Moreover, the cell apoptosis rates were calculated to 68.9% and 33.6% in the αM/MPEG-PCL nanomicelles and the αM group, respectively, after 24 h treatment by a concentration of 12.5 μg/mL. Obviously, the αM/MPEG-PCL nanomicelles possessed more pronounced effect on the A375 cell apoptosis induction compared to the αM.

To explore the reason that the αM/MPEG-PCL micelles could enhance apoptosis induction, we compared the cellular uptake ability of the αM/MPEG-PCL nanomicelles and the αM in A375 cells. The HPLC assay results in Fig. 6b clearly demonstrated the αM/MPEG-PCL nanomicelles had a better cellular uptake behavior than the αM.

Since, the loss of mitochondrial potential is a significant event in the intrinsic apoptotic pathway, the mitochondrial membrane potential was performed for an investigation on the apoptotic pathway activated by the αM/MPEG-PCL nanomicelles. The Fig. 6c showed that the Rh123 accumulation in A375 and B16 cells treated with the αM/MPEG-PCL nanomicelles were cut down compared with the control. Indeed, the specific differences were presented as a concentration-dependent manner.

Additionally, several proteins-related mitochondrial apoptosis pathway were analyzed. After incubating with gradient concentrations of the αM/MPEG-PCL nanomicelles, A375 cells were lysed. The expression levels of Bax and Bcl-2 tested by western blot were summarized in Fig. 6d. The level of Bcl-2 was markedly reduced in a dose-dependent manner, while the level of Bax increased. Additionally, these results implied that the αM/MPEG-PCL nanomicelles-induced apoptosis in melanoma cells by the mitochondrial apoptosis pathway.

Moreover, the mitochondrial apoptosis pathway is a key step in the intrinsic apoptotic pathway which turns on the caspase cascade. Hence, we further examined the expression level of caspase-3 and caspase-9 in the A375 cells. As shown in Fig. 6e, pro-caspase-3 and pro-caspase-9 decreased significantly in the A375 cells exposed to the αM/MPEG-PCL nanomicelles. Simultaneously, the cleaved-caspase-3 and cleaved-caspase-9 increased in a dose-dependent manner.

Finally, we detected the expression quantity alteration of caspase-8, a performer in exogenous apoptosis pathway. As shown in Fig. 6f, pro-caspase-8 level was downregulated and the expression quantity of cleaved-caspase-8 was increased in A375 cells treated by the αM/MPEG-PCL nanomicelles.

The proliferation and migration caused by the αM/MPEG-PCL nanomicelles in the human umbilical vein endothelial (HUVEC) cells were also studied to explore the preliminary anti-angiogenic mechanism. As shown in Figure S1, the αM/MPEG-PCL nanomicelles inhibited the proliferation and suppressed the migration in a dose- and time-dependent manner.

Based on the above evaluations, we hypothesized that the αM/MPEG-PCL nanomicelles triggered apoptosis via both the mitochondrial-mediated intrinsic pathway and the exogenous apoptosis pathway. The αM/MPEG-PCL nanomicelles also exhibited potential anti-angiogenic effect.

### In vivo anti-cancer activity of the αM nanomicelles

**Fig. 7:**
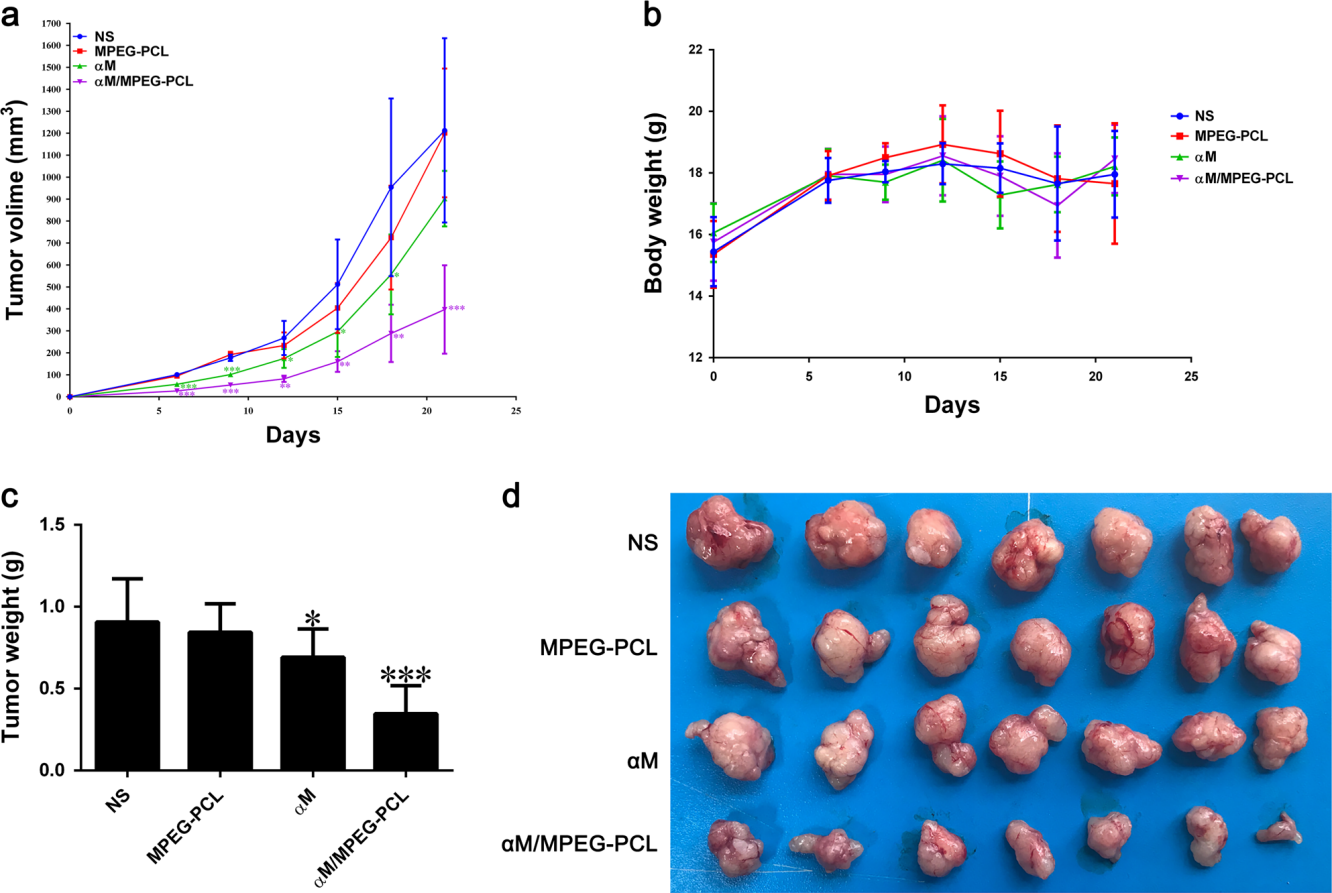
Anti-tumor effect of the αM/MPEG-PCL nanomicelles in vivo. **a** Nude mice were inoculated with A375 cells. After 7 days, the mice were randomly assigned to four groups treated, respectively, with the normal saline (NS), the empty MPEG-PCL nanomicelles (MPEG-PCL), the free αM or the αM/MPEG-PCL. Tumor development curve were measured on the indicated days. **b** The body weight in each treatment group on the indicated days. **c** The numerical statement of tumor weight in different groups on day 21. **d** Representative pictures of subcutaneous tumors in each treatment group on day 21. *P < 0.05; **P < 0.01; ***P < 0.001 compared to vehicle (NS group)

Mice subcutaneous melanoma cancer model was employed to evaluate the effect of the αM/MPEG-PCL nanomicelles on anti-tumor activity. Tumor-bearing mice were assigned casually to four groups (7 mice/group), and treated with the normal saline (NS), the MPEG-PCL, the αM, and the αM/MPEG-PCL nanomicelles, respectively, via intravenous injection. The body weight of mice and the volume of tumor were measured every 3 days. The tumor volume curves of each group were showed in Fig. 7a. The results indicated that the αM/MPEG-PCL nanomicelles possessed better effect on tumor growth inhibition compared to the free αM. Moreover, there was no obvious effect on the NS and MPEG-PCL groups. More specifically, the body weight and tumor weight of the four groups were presented in Fig. 7b and c, respectively, as well as the tumor photos demonstrated in Fig. 7d. We evidently observed that the group treated with the αM/MPEG-PCL nanomicelles held smaller tumor than those in the other groups. In conclusion, the strategy that encapsulated the αM into the MPEG-PCL nanomicelles comprehensively enhanced the anti-tumor activity of αM in vivo.

### Immunohistochemical analysis

**Fig. 8:**
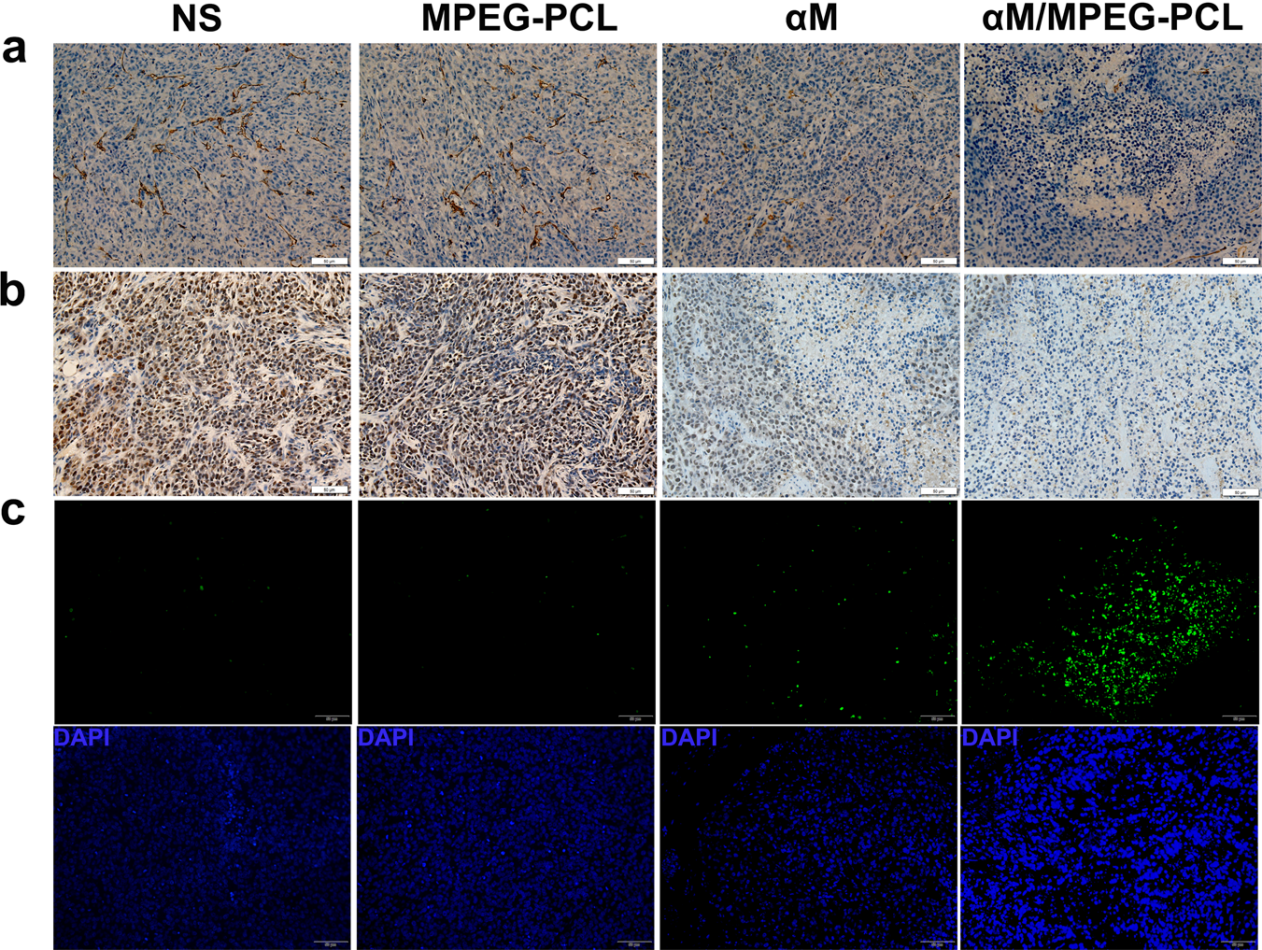
Immunohistochemistry staining of tumor sections for the research of mechanisms of anti-melanoma in vivo. **a** CD31 immunofluorescence staining assay. Tumor tissues sections from four groups treated with the normal saline (NS), the empty MEPG-PCL nanomicelles (MPEG-PCL), the free αM (αM), and the αM/MPEG-PCL nanomicelles (αM/MPEG-PCL) were subjected to immunohistochemistry to analyze anti-angiogenesis effect. **b** PCNA immunohistochemical staining analysis. Tumor sections from four groups (NS, MPEG-PCL, αM, αM/MPEG-PCL) were analyzed to PCNA immunohistochemical to detect tumor cell proliferation. **c** TUNEL assay. The apoptosis of A375 tumor sections was measured via TUNEL staining. The group treated with the αM/MPEG-PCL nanomicelles showed the most apoptotic cells, which demonstrated that the MPEG-PCL nanomicelles could improve the anti-tumor activity of the αM

In order to detect the effect of the αM/MPEG-PCL nanomicelles anti-angiogenesis in frozen tumor sections, we performed an immunohistochemical analysis using anti-CD31 monoclonal antibody. As demonstrated in Fig. 8a, CD31-positive endothelial cells in the group of the αM/MPEG-PCL nanomicelles were remarkably less than that of the αM, which was measured more CD31-positive endothelial cells than NS and MPEG-PCL nanomicelles groups.

Moreover, in order to confirm the effect of the αM/MPEG-PCL nanomicelles on the melanoma cell proliferation, we performed proliferating cell nuclear antigen (PCNA) stained. In short, the tumor sections of each group were stained with PCNA and examined under a microscope. The iconic images were shown in Fig. 8b. The PCNA-positive tumor cells in the group of αM/MPEG-PCL nanomicelles were markedly lower than the ones in other groups. The results suggested that the αM/MPEG-PCL nanomicelles significantly suppressed the proliferation of melanoma in vivo.

Simultaneously, we demonstrated that the αM/MPEG-PCL nanomicelles-induced melanoma cells apoptosis in vitro. In order to further validate the mechanisms in vivo and explore whether the nanomicelles enhanced the activity of the αM against melanoma, the tumor sections were detected a terminal-deoxynucleoitidyl transferase mediated nick end labeling (TUNEL) assay. As shown in Fig. 8c, it was extremely obvious that the tumor samples treated with the αM and the αM/MPEG-PCL nanomicelles existed more significant cellular apoptosis than other groups. In addition, there were more apoptosis cells in the αM/MPEG-PCL nanomicelles group than the αM group. The apoptosis index in the αM/MPEG-PCL nanomicelles group was more remarkable than the one in the αM group, MPEG-PCL group and NS group, which implied the αM/MPEG-PCL nanomicelles-induced stronger apoptosis of melanoma in vivo compared with the αM.

### Safety profile of the αM/MPEG-PCL nanomicelles

To further investigate the safety profile of the αM/MPEG-PCL nanomicelles, we performed several assays, including blood routine examination, blood chemistry test, and Hematoxylin-Eosin (HE) staining analysis. Obviously, no pathological change was found after the αM/MPEG-PCL nanomicelles treatment in Figure S2 and S3. Moreover, in the Figure S4, no evidentially pathological morphologic change was observed after the αM/MPEG-PCL nanomicelles therapy occurred in the lung, liver, heart, kidney, and spleen via microscopy study compared with the control group.

## Discussion

As a traditional and popular natural medicine, the αM has possessed promising application to treat various diseases^24,25^. Previous studies have shown that the αM offered good therapeutic effect on a series of cancers^22–24^. Nevertheless, the clinical application is gravely limited because of hydrophobicity. In this study, the MPEG-PCL nanomicelles were successfully applied in resolving the poor aqueous solubility of the αM and enhancing the activity of anti-melanoma.

Currently, nanometer material has been reported to be applied to heighten the solubility and strengthen the biological availability of hydrophobic drug. As a promising biodegradable nanometer material, the MPEG-PCL nanomicelles has been widely used to deliver drugs for tumor therapy owing to its outstanding drug entrapment efficiency (EE), high stability and hypotoxicity^12^. In this study, we developed the αM/MPEG-PCL nanomicelles via a one-step solvent evaporation way. The αM was wrapped in the MPEG-PCL nanomicelles to improve its water solubility, which obtained a better anti-melanoma activity. Furthermore, we compared with the free αM and the αM/MPEG-PCL nanomicelles on the effect of anti-melanoma as well as probed the mechanisms of action of the αM/MPEG-PCL nanomicelles.

In our survey, the αM/MPEG-PCL nanomicelles were provided with stable drug loading and high-encapsulation efficiency. Meanwhile, the characterization of the αM/MPEG-PCL nanomicelles illustrated small particle size and nearly neutral surface charge (zeta potential = −2.1 mv), as well as monodisperse suspension in aqueous phase owing to the core-shell structure of polymeric nanomicelles. In addition, the nanostructure of the nanomicelles was monitored by TEM, which implied that the prepared αM/MPEG-PCL nanomicelles were solvable and stable in aqueous solution. In previous studies, nanomicelles, permeability, and retention (EPR) effect were enhanced if the drug-loaded nanomicelles possessed suitable size (10–100 nm), and consequently, the accumulation in cancer tissue increased anti-carcinoma effects^26,27^. Besides, the release behavior of the presented highly sustained drug was realized in the αM nanomicelles owing to the tiny size and the stable hydrophilic interface of the MPEG-PCL nanomicelles. Furthermore, the results of drug-release experiments in vitro illustrated the MPEG-PCL nanomicelles prolonged the duration of the αM release. Meanwhile, in the study of pharmacokinetics analysis in vivo, the plasma concentrations of the αM/MPEG-PCL nanomicelles group markedly reduced than the αM groups. The αM/MPEG-PCL nanomicelles were obviously observed that it extended the αM treating duration to improve the effectiveness by sustaining release. The results indicated the αM/MPEG-PCL nanomicelles possessed excellent anti-neoplastic potentials.

Meanwhile, a series of experiments were performed to assess the anti-melanoma effect of the αM/MPEG-PCL nanomicelles compared with the free αM, including both in vitro and in vivo assays. We hypothesized some anti-melanoma mechanisms for the αM/MPEG-PCL nanomicelles. First, in the cytotoxicity tests, we found the αM/MPEG-PCL nanomicelles showed a better inhibitory effect on the growth and proliferation of the melanoma cells (A375 and B16 cells) than the free αM via MTT assay and colony formation experiments. Meanwhile, the αM/MPEG-PCL nanomicelles showed a lower toxicity in the non-tumor cell lines, including HEK293T, LO2, and Vero cells, compared with the melanoma cells. Moreover, the αM/MPEG-PCL nanomicelles induced the apoptosis in the melanoma cells via FCM assay. The above results demonstrated the αM/MPEG-PCL nanomicelles had more apoptosis ratio than the free αM. In addition, we found the αM with the encapsulation of the MPEG-PCL nanomicelles was more easily absorbed by melanoma cells, which contributed to achieving a higher concentration in cellular. Hence, we hypothesized that the enhancement effect of anti-melanoma activity was derived from the high uptake ratio of the αM/MPEG-PCL nanomicelles in vitro.

These results suggested the αM/MPEG-PCL nanomicelles suppressed melanoma cells growth retardation associated with apoptosis-inducing process. It is well-known that apoptosis plays a vital role in the development and maintenance of tissue homeostasis as well as is indispensable function for the initiation and progression of cancer^28–30^, including the mitochondrial (intrinsic) and death receptor pathways (extrinsic)^31^. In the process of exploring the potential mechanisms of the αM/MPEG-PCL nanomicelles-induced apoptosis in melanoma, we found the variation of mitochondrial membrane potential (MMP) with Rh123 stained and the expression of a series of apoptosis-associated proteins with WB. Experimental results validated the αM/MPEG-PCL nanomicelles significantly declined the MMP, which is one of the major characteristics of the mitochondrial-induced apoptosis pathway^32^. Moreover, the αM/MPEG-PCL nanomicelles enhanced the expression of Bax (pro-apoptotic protein) while decreased the expression of Bcl-2 (anti-apoptotic protein)^33^. Furthermore, we found the reduction in the expression of pro-caspase-9 and pro-caspase-8 as well as the augment with the expression of cleaved-caspase-9 and cleaved-caspase-8 in WB experiments. More importantly, after incubation with the αM/MPEG-PCL nanomicelles, the downregulated expression of pro-caspase-3 and the upregulated expression of cleaved-caspase-3 were observed in A375 cells. It is well-known that caspase-3, an executioner caspase^34^, is the end of the stage of apoptosis commencer shared by the both pathways^35,36^. Consequently, our research indicated that the αM/MPEG-PCL nanomicelles induced apoptosis process in the melanoma cells via mitochondrial-mediated endogenous pathway and extrinsic apoptosis pathway. In the nude mouse model injected A375 cells, our results demonstrated that the weights and volumes of tumors were more significantly suppressed in the αM/MPEG-PCL nanomicelles group compared with any another group. Simultaneously, no obvious changes on the body weight of nude mice were found, which indicated the αM/MPEG-PCL nanomicelles had stronger anti-melanoma effect and exhibited low cytotoxicity.

Furthermore, the possible mechanisms of the anti-melanoma activity of the αM/MPEG-PCL nanomicelles in vivo were proposed. It was reported that the expression of PCNA was closely related to the proliferation^37^. As shown in Fig. 8b, the PCNA-positive cells in the αM/MPEG-PCL nanomicelles group were much fewer than the ones in the other groups, suggesting the αM/MPEG-PCL nanomicelles blocked melanoma associated with cell proliferation suppression and possessed better effect compared to the free αM. Furthermore, angiogenesis is crucial in tumor growth and anti-angiogenic, which is considered as a promising therapeutic strategy for tumor treatment^38,39^. Hence, we implemented the immunohistochemical analysis of CD31. The results showed that through the αM/MPEG-PCL nanomicelles treatment presented fewer angiogenesis effects than the free αM, NS, and MPEG-PCL groups in tumor tissue, which indeed proved the αM/MPEG-PCL nanomicelles exerted the anti-melanoma effect involving in the distinct impairment of neovascularization. Indeed, the αM/MPEG-PCL nanomicelles could possess the inhibition on cell viability and cell migration of the HUVEC cells in vitro. Additionally, the apoptosis tumor cells were detected with TUNEL staining in vivo and showed in Fig. 8c. On the basis of the results, the αM/MPEG-PCL nanomicelles induced a higher apoptosis ratio than the free αM.

In conclusion, the αM/MPEG-PCL nanomicelles were developed by a thin-film hydration method and applied to the therapy of melanoma in vitro and in vivo. The αM/MPEG-PCL nanomicelles with a high EE and controlled release behavior in vitro possessed more excellent cellular uptake, cytotoxicity, proliferation inhibition, and apoptosis-inducing in the melanoma cells than the free αM. Moreover, a slow excretion behavior from blood vessels was also observed in the αM/MPEG-PCL nanomicelles group. Besides, in A375 cell-bearing tumor model, the αM/MPEG-PCL nanomicelles more dramatically inhibited tumor growth and angiogenesis than other groups (including the free αM). In the exploration of mechanisms, we found the αM/MPEG-PCL nanomicelles repressed the melanoma cells via the mitochondrial-mediated intrinsic pathway and the exogenous apoptosis pathway. In short, the αM/MPEG-PCL nanomicelles presented in this work demonstrated an improved anti-melanoma activity both in vitro and in vivo, which suggested this novel formulation had potential applications in the melanoma therapy.

## Methods

### Preparation of αM/MPEG-PCL nanomicelles

For preparation of the αM/MPEG-PCL nanomicelles, 10 mg αM and 90 mg MPEG-PCL copolymer were re-suspended in 10 mL methanol with suitably stirring. Subsequently, the methanol was removed by a rotary evaporator at 50 °C. During the process, an amorphous substance was obtained including the αM and the MPEG-PCL. Meanwhile, the αM was dissolved into MPEG-PCL copolymer to get an amorphous substance. Then, nanomicellesthe co-evaporation was dissolved in Milli-Q water at 55 °C to achieve the αM/MPEG-PCL nanomicelles by self-assembly method. Finally, the produce of the αM/MPEG-PCL nanomicelles solution with a concentration of 5 mg/mL was filtered by 0.22 μm syringe filter (Jinteng, Tianjin, China), lyophilized and stored at 4 °C for future use.

### Molecular modeling study of the interaction between the αM and the copolymer MPEG-PCL

The interaction between the αM and the copolymer MPEG-PCL was simulated. First, the 3D structures of the αM and the copolymer were fabricated by the same ways as described in previous studies ^12^. After that, the αM was docked randomly to the simulated copolymer MPEG-PCL by merging the αM to the copolymer in the workspace of HyperChem. After that, the αM/MPEG-PCL complex experienced 2 stage Langevin Dynamics simulation.

At the first stage of simulation, the interaction between the αM and the copolymer MPEG-PCL was simulated, and the solvation effect was considered implicitly by setting the scale factor for the dielectric permittivity to 80. At the second stage, the complex was further simulated in aqueous solution and in tumor tissue, respectively. The interaction between the αM and the MPEG-PCL near the tumor tissue was simulated by setting the scale factor to 26. At each stage, the running time was set to 100 ps and the temperature, friction coefficient, and random seed were set to 300 K, 0.075 ps^−1^, and 0, respectively. CHARMM27 was chosen as the force field.

### In vitro release

Release behaviors of the free αM and the αM/MPEG-PCL nanomicelles were evaluated by dialysis with a molecular cutoff 3.5 KDa. Briefly, 1 mL drug solution (the free αM or the αM/MPEG-PCL nanomicelles) was transferred in dialysis bags suspended in in 40 mL PBS (pH = 7.4) containing Tween 80 (0.5%, w/w) at 37 °C. (Sigma-Aldrich Co.). The whole device was placed in a shaking water with gentle shaking (100 rpm). After that, at predetermined time intervals, 200 μL release medium samples were collected and supplemented with an equal volume of fresh medium. The amount of released αM was quantitated by HPLC. The experiment was repeated three times and the results were expressed as mean ± SD.

### Pharmacokinetic

The Sprague Dawley (SD) rats were randomly divided into two groups: the free αM and the αM/MPEG-PCL nanomicelles. Both groups were, respectively, dissolved into the mixed solution containing Tween 80 and dehydrated alcohol (1:1, v/v) and the normal saline (NS). After starved overnight, the rats received a dose of 50 mg/kg drugs administered intravenously. Then, the blood was collected by a capillary from eye sockets at various predetermined intervals (5 min, 15 min, 30 min, 45 min, 1 h, 2 h, 4 h, 6 h, and 8 h; three mice each time point). The blood samples were extracted by methanol, evaporated, and stored at −20 °C until LC–MS analysis.

### Cell proliferation

A375, B16, and HUVEC cells were seeded in 96-well plates at 2.5–5 × 10^3^ cells/well. Gradient concentrations of the αM and the αM/MPEG-PCL nanomicelles were added to the plates for specific time. Twenty microliters of MTT working solution (5 mg/mL) was added to each well. After 4 h incubation at 37 °C, the supernatant was removed and 150 μL dimethyl sulfoxide (DMSO) per well was appended. The plates laid up a shaker for about 10 min. The optical density was measured via Spectra MAX M5 microplate spectrophotometer at 570 nm.

### Colony formation assay

A total of 300–500 cells (A375 or B16 cells) were plated in a 6-well plate. After 3 days incubation, the cells were treated with diverse concentrations of the αM and the αM/MPEG-PCL nanomicelles for 15 days. The culture medium was replaced with a fresh medium containing same concentrations of drug every 3 days. After colonies were formed, the cells were stained with crystal violet and fixed by methanol.

### Cells apoptotic assay by FCM

The A375 and B16 cells were treated with the αM and the αM/MPEG-PCL for 24 h and collected in the flow tubes. After adding the PI and Annexin V into the flow tubes in accordance with the usage instructions, we detected the level of apoptosis via FCM (BD Bioscience). Finally, the FlowJo software was used to analyze the data that represented the average of independent experiments.

### Examination of mitochondrial membrane potential (Ψm)

A375 and B16 cells were treated with indicated doses of the αM/MPEG-PCL for 24 h incubated with 5 µg/mL Rhodamine 123 (Rh123) at 37 °C in the dark for 30 min. The stained cells were collected and washed twice with cold PBS, and then the fluorescence was subsequently detected by FCM.

### Cellular uptake

A375 cells were grown in complete medium to reach 80% confluence. After removing the original medium, the cells were treated with the αM (5 μg/mL, 10 μg/mL) and the αM/MPEG-PCL (5 μg/mL, 10 μg/mL) for 3 h. The cells were collected and washed for three times by PBS. Then the cells were re-suspended in methanol and sonicated to collect the drug from cellular. The supernatants were collected after twice centrifugation (13,000 rpm, 10 min). Finally, the drug content was determined by HPLC. The test was performed in triplicate.

### Western blot analysis

A375 cells treated with various concentrations of the αM/MPEG-PCL were lysed in RIPA buffer (Beyotime, Beijing, China). The total proteins were quantified by Nanodrop™ 2000/2000c spectrophotometers (Thermo Fisher Scientific, Waltham, Massachusetts, USA) and equalized before loading. Equal amounts of protein from each sample were fractionated by sodium dodecyl sulphate polyacrylamide gel (SDS-PAGE) electrophoresis and transferred onto polyvinylidene difluoride (PVDF) membrane accordingly. Then, the membranes were blocked with 5% bovine serum albumin (BSA) for 2 h at 37 °C and incubated with homologous primary antibody working solution overnight at 4 °C. After washed three times (15 min per time) by tris-buffered saline with Tween 20 (TBST), the membranes were incubated with corresponding secondary antibody with a working concentration based on its specification at 37 °C for 1 h. Finally, the spots of proteins were measured via an enhanced chemiluminescence system (Amersham Biosciences, Buckinghamshire, UK). β-actin was regarded as a reference.

### Evaluation of the anti-melanoma effect of the αM/MPEG-PCL nanomicelles in vivo

A375 cells (1 × 10^7^) in 0.1 mL serum-free medium were injected subcutaneously into the female BALB/c athymic nude mice. Mice were randomly assigned into four groups (7 mice/group) when the tumor volume reached ~100 mm^3^. After that, the four groups were treated with the NS, the MPEG-PCL, the free αM (50 mg/kg) or the αM/MPEG-PCL (50 mg/kg), respectively. The tumor volumes were measured every 3 days with a vernier caliper and tumor volumes were calculated according to the equation: *V* = *L* × *W*^2^ × 0.52. Moreover, the body weight of each mouse was measured every 3 days and treatment conditions were observed daily. At the termination of the experiment, mice were killed by cervical dislocation. In addition, tumor samples were collected and weighed.

### TUNEL assay

The tumors were fixed in PBS containing 4% paraformaldehyde for at least 24 h, following by exposure in 70% ethanol overnight and embedded in paraffin. The sections (3–5 μm thick) were cut and mounted. A commercially procurable terminal-deoxynucleoitidyl transferase mediated nick end labeling (TUNEL, Promega, Madison, WI, USA) was applied to detect apoptotic cells within A375 tumor cells. The TUNEL assay was carried out according to the manufacturer’s protocol and the samples were examined with a fluorescence microscope (×400).

### Immunohistochemical (IHC) determination of PCNA and CD31

The expression of PCNA and CD31 were estimated by immunochemistry with PCNA and CD31 antibodies. Briefly, the frozen tumor tissues were fixed in acetone and washed twice with PBS. Following, the handled samples were stained with rabbit anti-mouse CD31 monoclonal antibody (1:50; Abcam, Cambridge, UK) and rabbit anti-rat PCNA polyclonal antibody (1:50; BD Pharmingen^TM^; BD Biosciences, San Jose, CA, USA). After washing twice with PBS, samples were stained with secondary antibody combining with FITC or Rhodamine. Finally, we surveyed the cells possessing-positive property under microscope (×400) and counted automatically the number of capillaries in 5 randomly selected fields.

### Statistical analysis

Data of three-independent experiments were expressed as the mean ± standard deviation (SD). Statistical analysis was performed with Graph Pad and Excel. Student’s test was employed for comparing treatment and control groups. *P*-values were marked as follows: *P < 0.05; **P < 0.01; ***P < 0.001.

## Supplementary information

Figure S1

Figure S2

Figure S3

Figure S4

Supporting information
