# Supplementary figures and images for: Applying an innovative biodegradable self-assembly nanomicelles to deliver α-mangostin for improving anti-melanoma activity
Source: Cell Death Dis. 2019 Feb 15;10(3):146. doi: 10.1038/s41419-019-1323-9 (PMC6377678; doi:10.1038/s41419-019-1323-9)

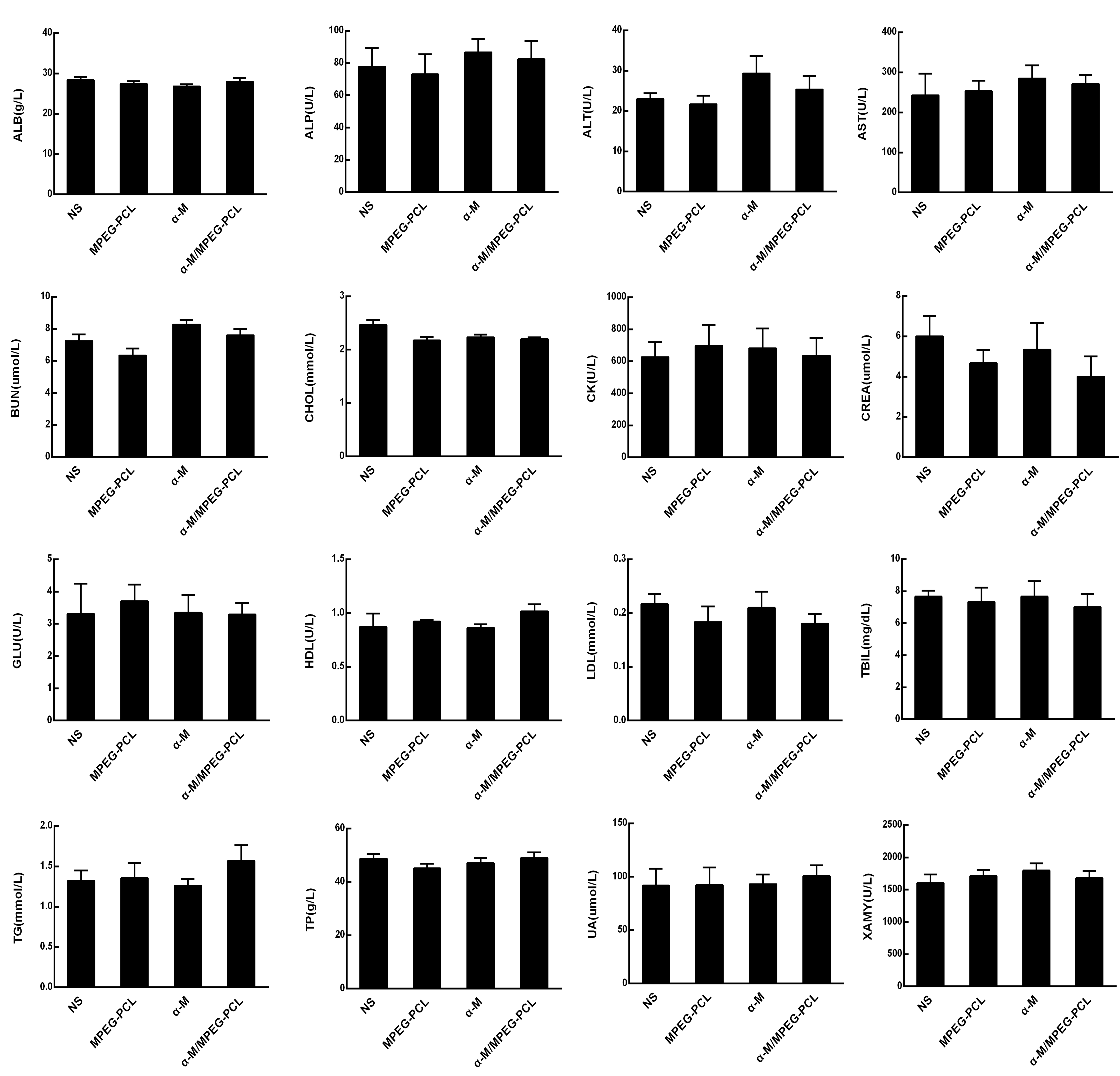

Supplement: Supplementary file 2 — Figure S2 [file 41419_2019_1323_MOESM2_ESM.tif]

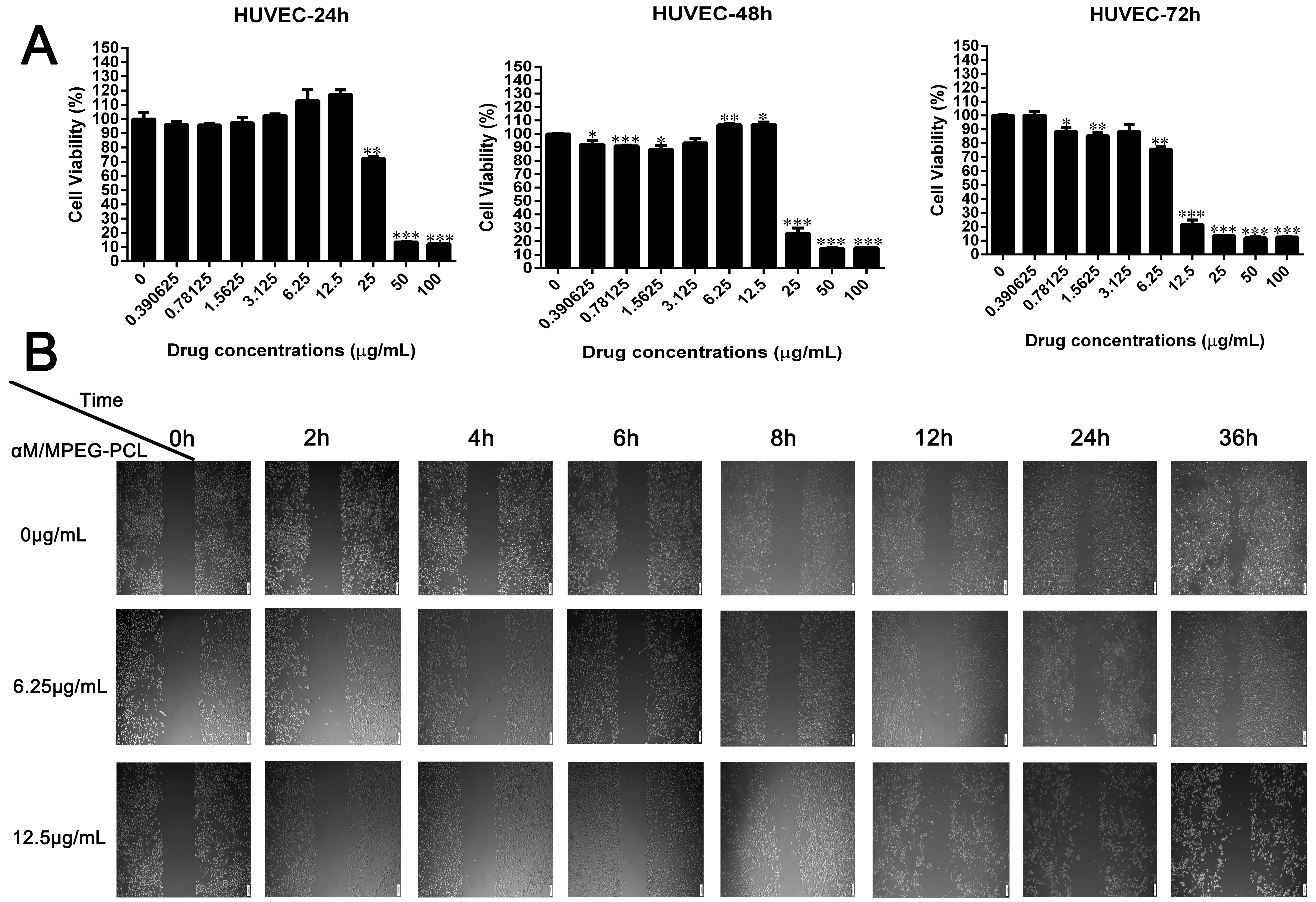

Supplement: Supplementary file 4 — Figure S4 [file 41419_2019_1323_MOESM4_ESM.tif]
