# Supplementary material for: Applying an innovative biodegradable self-assembly nanomicelles to deliver α-mangostin for improving anti-melanoma activity
Source: Cell Death Dis. 2019 Feb 15;10(3):146. doi: 10.1038/s41419-019-1323-9 (PMC6377678; doi:10.1038/s41419-019-1323-9)
Supplement: Supplementary file 5 — Supporting information [file 41419_2019_1323_MOESM5_ESM.doc]

**Applying an innovative** **biodegradable self-assembly nanomicelles to deliver α-mangostin for improving anti-melanoma activity**

Shuping Yang^1,*^, Xiang Gao^1,*,#^, Yihong He^1^, Yuzhu Hu^1^, Bocheng Xu^1^, Zhiqiang Cheng^2^, Mingli Xiang^1,#^, Yongmei Xie^1,#^

^1^ Department of Neurosurgery and Institute of Neurosurgery, State Key Lab of Biotherapy and Cancer Center, West China Hospital, Sichuan University and Collaborative Innovation Center for Biotherapy, Chengdu, 610041, PR China.

^2^ Department of Pharmacology and Molecular Sciences, Johns Hopkins University School of Medicine, Baltimore, MD, 21205, USA.

* These authors contributed equally to this work.

^#^Correspondence: Xiang Gao, Mingli Xiang, Yongmei Xie.

Tel: +86 28 8542 2136; Fax: +86 28 8550 2796

Email: [xianggao@scu.edu.cn](mailto:xianggao@scu.edu.cn);

[xiang](mailto:xiang) mingli@scu.edu.cn;

xieym@scu.edu.cn.

**Abbreviations:** **DMSO**, dimethyl sulfoxide; **PH**, potential of hydrogen; **PBS**, phosphate-buffered saline; **FBS**, fetal bovine serum; **MTT**, 3-(4,5-dimethylthiazol-2-yl)-2,5-diphenyltetrazolium bromide; **HPLC**, high-performance liquid chromatography; **FCM**, flow cytometry; **DCFH-DA**, 2’,7’-dichlorodihydrofluorescein diacetate; **Rh123**, rhodamine123; **PI**, propidium iodide; **WB**, western blot; **BSA**, bovine serum albumin; **SDS-PAGE**, sodium dodecyl sulfate-polyacrylamide gel electrophoresis; **PVDF**, polyvinylidene difluoride; **NS**, normal saline; **IHC**, immunohistochemical; **H&E**, hematoxylin and eosin.

**Methods**

The αM was obtained from Chengdu Biopurify Phytochemicals Ltd and the structure was confirmed by ^1^H NMR. 2-(6-Amino-3-imino-3H-xant-hen-9-yl) benzoic acid methyl ester (Rh123) and 3-(4,5-dimethylthiazol-2-yl)-2,5-di-phenyltetrazolium bromide (MTT) were purchased from Sigma Chemical Co. (St Louis, MO, USA). The Annexin V-FITC and PI Apoptosis Detection Kit was purchased from KeyGen Biotech (Nanjing, China). The primary antibodies against Bax (Item No. 2772s), cleaved-caspase3 (Item No. 9661s), caspase3 (Item No. 9662s), cleaved-caspase8 (Item No. 8592s), casepase8 (Item No. 4790s), cleaved-caspase9 (Item No. 9507s), caspase9 (Item No. 9506s) were purchased from Cell Signaling Technology (Beverly, MA, USA). Bcl-2 (Item No. ab59348) and β-actin (Item No. ab8226) were obtained from Abcam (Cambridge, UK). The secondary antibodies derived from Zhongshan Biological (Beijing, China). Methanol (high-performance liquid chromatography [HPLC] grade) was purchased from Kelong Chemicals (Chengdu, China). Dimethyl sulfoxide (DMSO) was purchased from Sigma Chemical Co. (St Louis, MO, USA).

In our study, seven-week-old female BALB/c athymic nude mice were purchased from the Laboratory Animal Center of Sichuan University. In addition, in animal procedures, we followed close to the agreement of provided by the Institutional Animal Care and Treatment Committee of Sichuan University (Chengdu, P.R. China). All mice received humanitarian treatment throughout the experiment period.

To make a stock solution at a concentration of 100 mg/mL, 10 mg αM was dissolved in 100 μL DMSO and then stored at -20 ℃. Mixed the stock solution and the relevant assay medium as dilution ratio 1:1000 in order to get the working solution, 0.1% DMSO (v/v) served as a control at the same time.

**Cell culture**

Cell lines (A375, B16, HEK293T, Vero and HUEVC cells) were obtained from the American Type Culture Collection (ATCC, Manassas, VA). LO2 cells was purchased from Shanghai Institute of Biochemistry and Cell Biology (Shanghai, China). A375 cells, B16 (B16-F10) cells, HEK293T cells, LO2 cells, and Vero cells were maintained in Dulbecco’s modified eagle medium (DMEM, Gibco, USA), supplemented with 10% fetal bovine serum (FBS, Gibco, Auckland, N.Z.), 100 U/mL penicillin and streptomycin. HUVEC cells were cultured in Roswell Park Memorial Institute-1640 (RPMI-1640, Gibco, USA), the other conditions were the same as the above. All cells were placed incubator at 37 ℃ in a humidified atmosphere of 5% CO_2_.

**Characterization of αM/MPEG-PCL nanomicelles**

Drug loading (DL) and encapsulation efficiency (EE) of αM/MPEG-PCL nanomicelles were determined as follows. Brieﬂy, 10 mg of lyophilized αM/MPEG-PCL nanomicelles were dissolved into 0.1 mL of methanol, and then, the solution was centrifuged at 13,000 rpm for 5 min to harvest the supernatant. The concentration of αM was determined via high performance liquid chromatography (HPLC, LC-20AD; Shimadzu Corporation, Tokyo, Japan). The EE and DL were calculated by the following equations:

$$\begin{matrix} \text{EE}=\frac{\text{Experimental drug loading}}{\text{Theoretical drug loading}}\times\text{100\% (1)} \\ \text{DL}=\frac{\text{Drug}}{\text{Polymer}+\text{drug}}\times\text{100\% (2)} \end{matrix}$$

The particle size and zeta potential of αM/MPEG-PCL nanomicelles were measured by dynamic light scattering (Malvern Nano-ZS 90). The 100 μL micelles were dissolved in 2 mL of distilled water (nanomicelles concentration: 1 mg/mL). All of the data were obtained from three independent experiments.

The following procedures, we detected the transmission electron microscopy (TEM) (H-6009IV, Hitachi, Japan). Briefly, the αM/MPEG-PCL nanomicelles solution was placed dropwise onto a copper grid. About 15 min after nanoparticle deposition, the grid was tapped with filter paper to get rid of excess water and stained with a solution of phosphotungstate acid (2%, w/v) for 20 min. TEM sample were obtained when the stained sample was allowed to air dry. A photomicrograph of the αM/MPEG-PCL micelles was obtained via using a transmission electron microscope (high-resolution transmission electron microscopy) (Tecnai G2 F20 S-TWIN).

### Endothelial cell migration assay

HUVEC cells were scratched via a sterile pipette tip through the cell monolayer in a 6-well plates when reaching 80% confluence. The original medium was replaced with fresh various concentrations of the αM/MPEG-PCL nanomicelles. After certain time point incubation under 37 ℃ and 5% CO_2_, cells were photographed with a microscope (Zeiss, Axiovert 200, Germany).

**Safety evaluation assay**

To investigate the safety profile in rats during the treatment, the blood was obtained from eyeball and mouse tissue samples (heart, liver, spleen, lung and kidney) were collected when the mice were sacrificed. The blood samples were used for blood routine analysis and blood chemistry analysis, meanwhile the tissue samples were fixed in 4% paraformaldehyde and embedded in paraffin. The following, the processed tissue samples were cut into 4-μm-thick sections and were stained with hematoxylin and eosin (H&E) for histopathologic examination.

**Supplemental Figures**


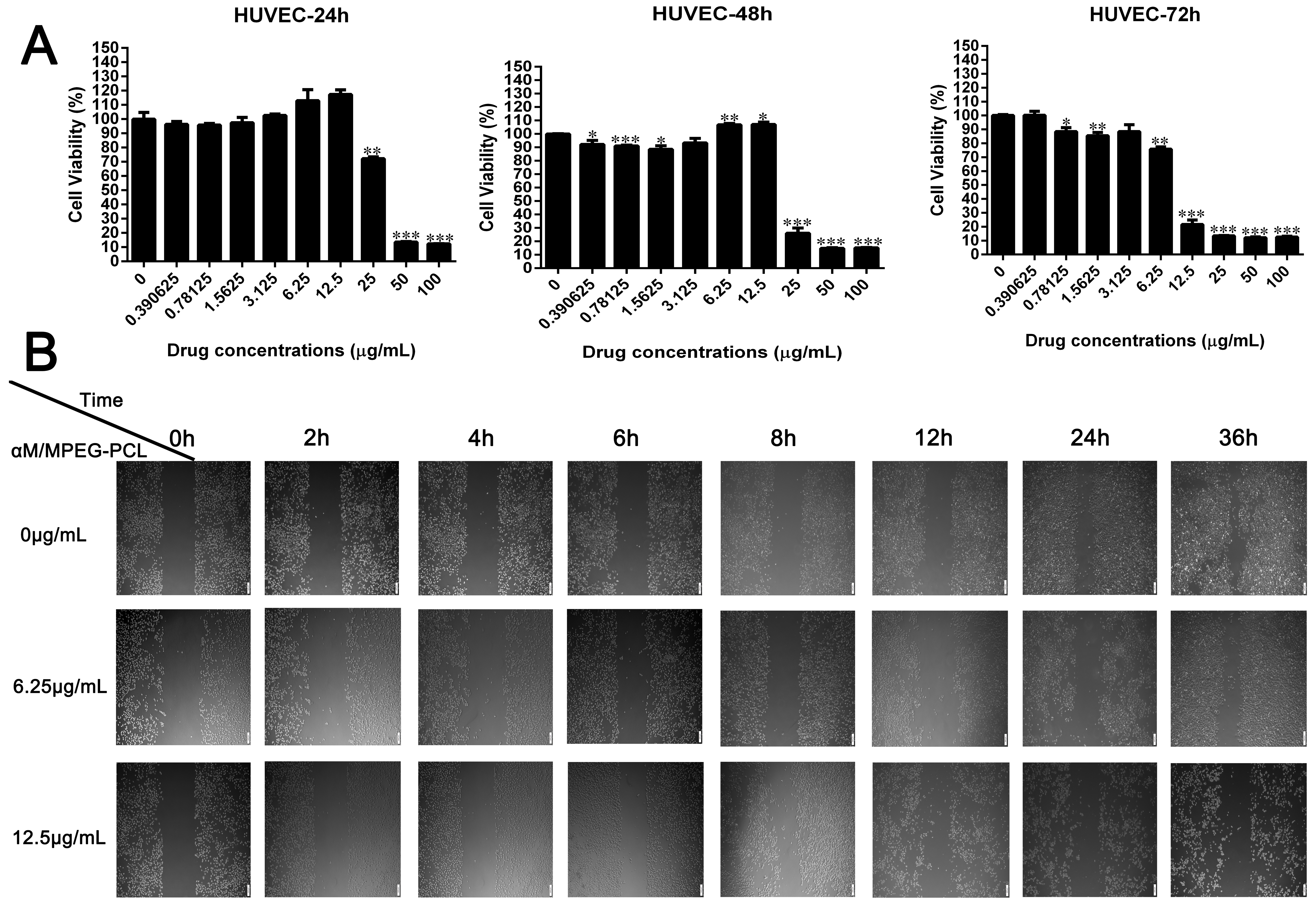


Figure S1. The effect of the αM/MPEG-PCL nanomicelles on cell viability and cell migration on HUVEC cells. (A) HUVEC cells were incubated with a series of concentrations of the αM/MPEG-PCL micelles for 48 h. Cell viability was evaluated with MTT assay. (B) HUVEC cells were seeded in six-well plates. Scratched a “wound” when the cells grew about 80% confluence. After treating with vehicle or the αM/MPEG-PCL nanomicelles (6.25 μg/mL and 12.5 μg/mL), the HUVEC cells were photographed (4 ×) at specific time.


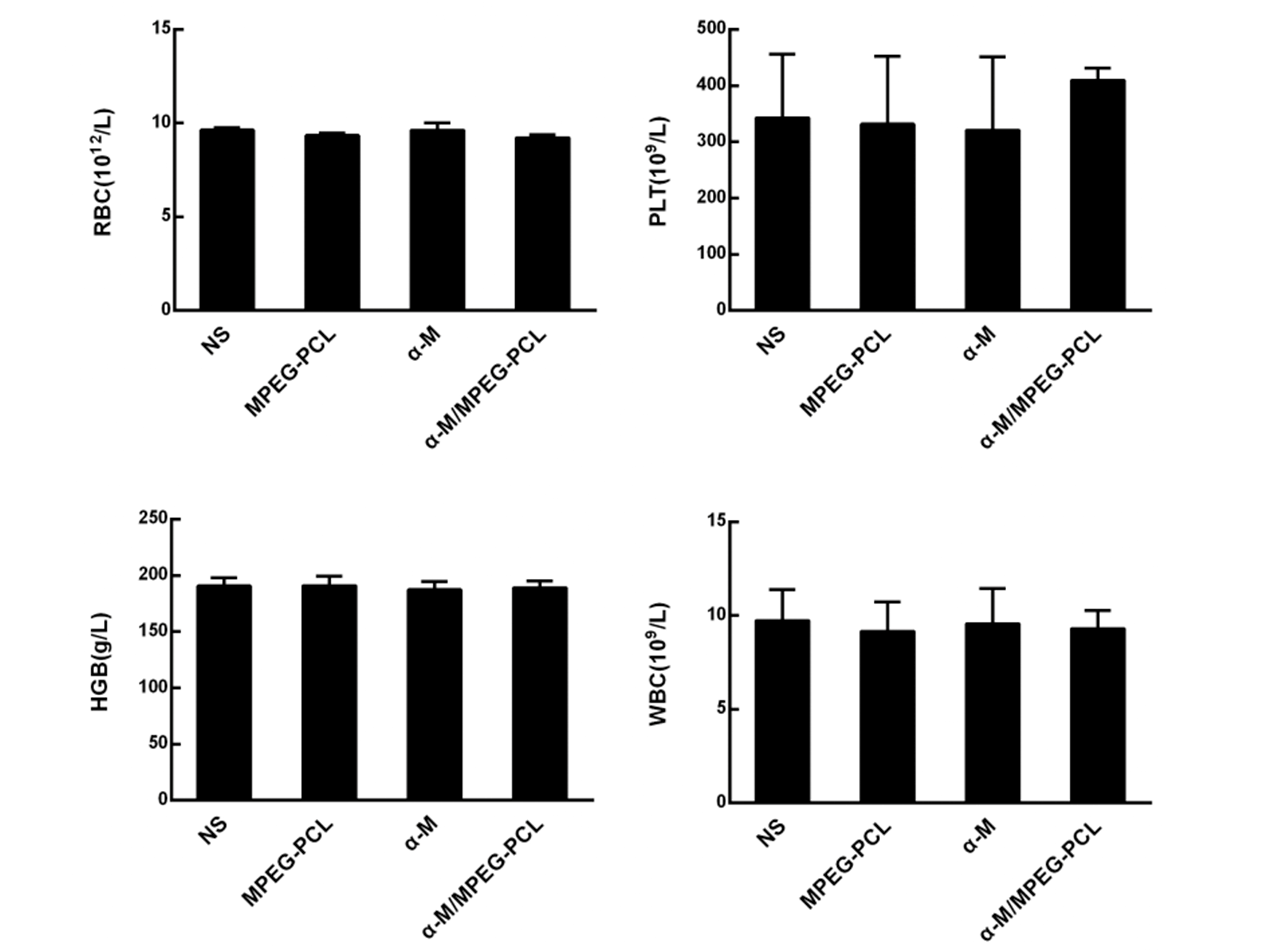


Figure S2. Routine analysis of blood of nude mice treated with NS, blank MPEG-PCL, the αM and the αM/MPEG-PCL nanomicelles.


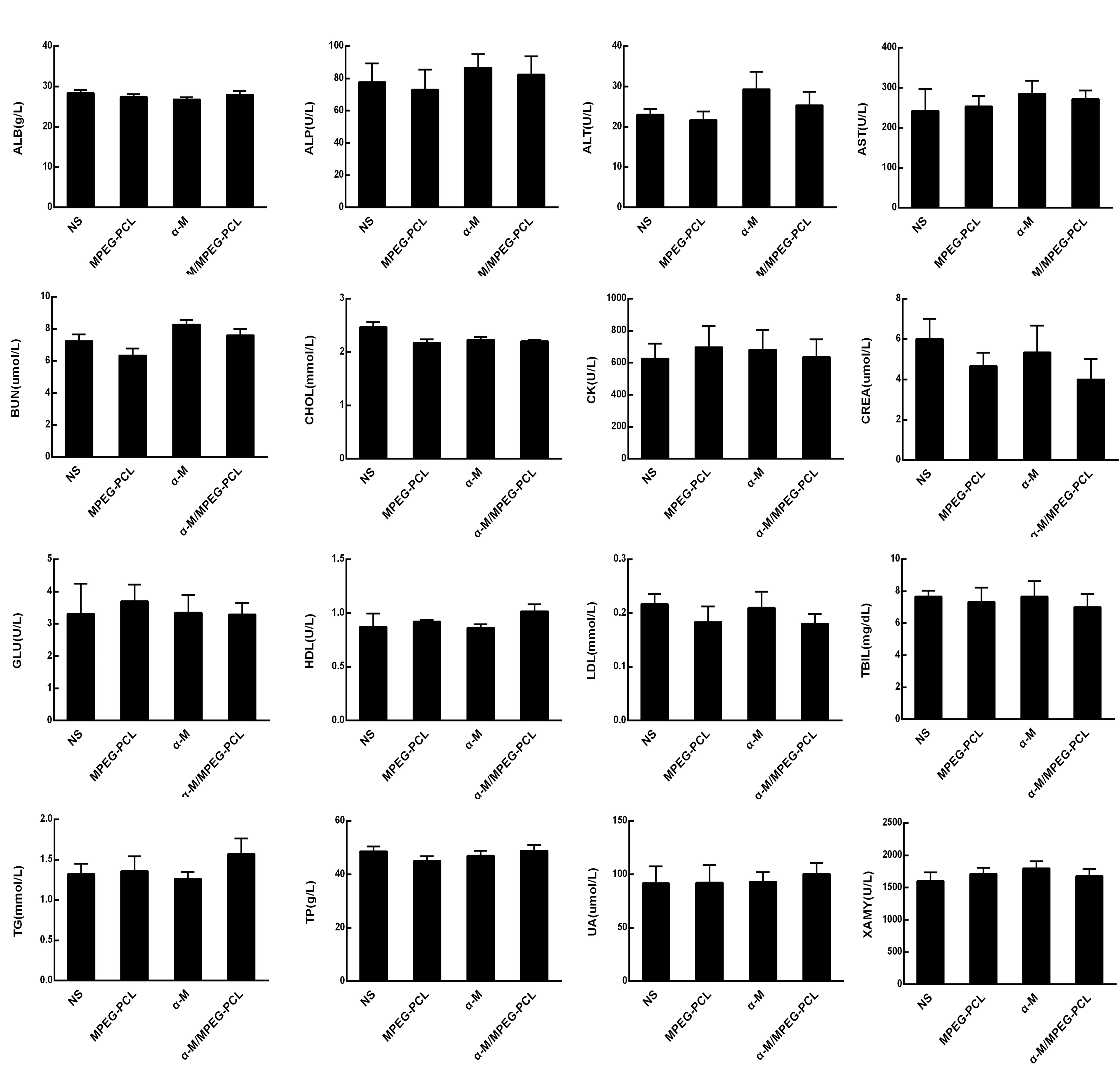


Figure S3. Blood biochemistry level of nude mice treated with NS, blank MPEG-PCL, the αM and the αM/MPEG-PCL nanomicelles.


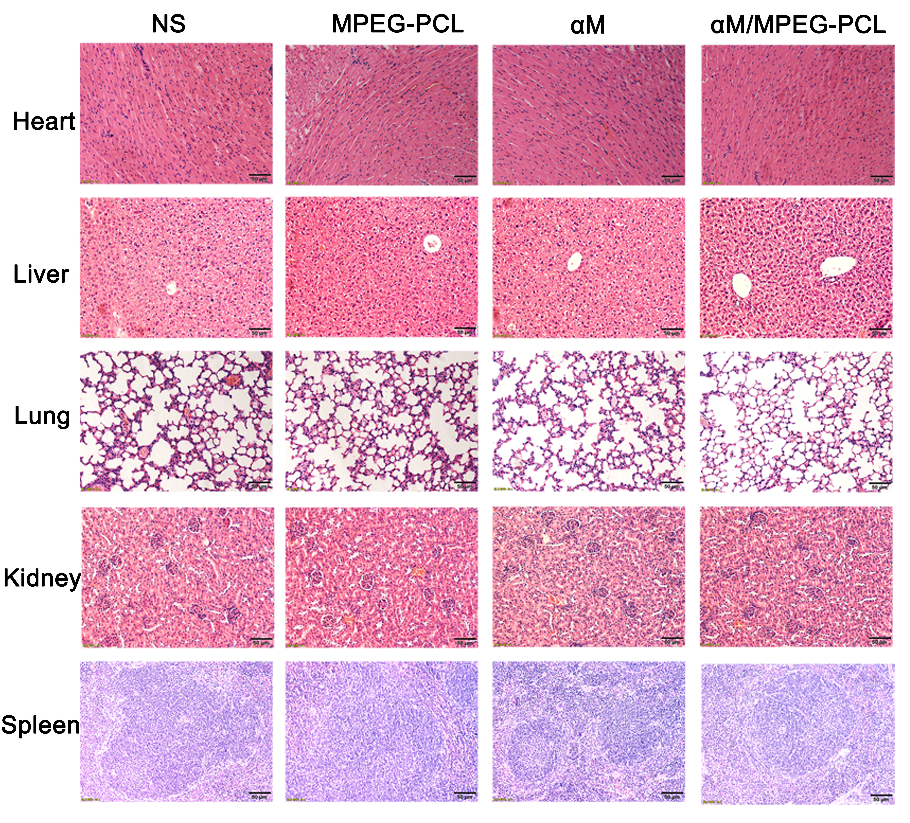


Figure S4. Evaluation of side effects on different organs (heart, liver, lung, kidney and spleen) of nude mice treated with NS, blank MPEG-PCL, the αM and the αM/MPEG-PCL nanomicelles by H&E staining.
